# Seasonal patterns of hip fracture incidence and mortality rates across age groups of older adults in Israel

**DOI:** 10.1007/s00068-024-02569-w

**Published:** 2024-07-25

**Authors:** Yaniv Yonai, Salim Masarwa, Merav Ben Natan, Yaron Berkovich

**Affiliations:** 1 The Orthopedics B Department, Hillel Yaffe Medical Center, Hadera, Israel; 2 Pat Matthews Academic School of Nursing, Hillel Yaffe Medical Center, P.O.B. 169, Hadera, 38100 Israel

**Keywords:** Hip fracture, Seasonality, Mortality

## Abstract

**Purpose:**

This study investigates the seasonal patterns of hip fracture occurrence and mortality rates in the elderly population of Israel.

**Methods:**

In this retrospective study, we examined a random sample of 400 patients who underwent a hip fracture repair surgery at a 495-bed hospital in northern-central Israel during the years 2021–2022.

**Results:**

Our findings reveal a heightened incidence of hip fractures during the spring (30.8%) in contrast to relatively consistent rates during other seasons (22-24.2%). Patients experiencing hip fractures in the spring were notably younger and had shorter hospital stays compared to those in other seasons. Furthermore, we identified seasonal variations in hip fracture incidence concerning gender, culture, and nationality. Over the 2-year follow-up period, 20% of patients had succumbed to mortality. The highest survival rate was associated with hip fractures sustained in the spring, while the lowest rates were observed in the autumn and winter.

**Conclusion:**

While our study highlights significant seasonal variations in hip fracture occurrence and mortality rates among the elderly population in Israel, caution is warranted in interpreting the implications for post-fracture care and resource allocation. The observed heightened incidence of hip fractures during the spring, particularly among younger patients with shorter hospital stays, suggests the need for further investigation into potential risk factors and preventive measures specific to this season. Additionally, our identification of seasonal variations in hip fracture incidence across demographic factors underscores the importance of tailored interventions to address the diverse needs of different populations.

## Introduction

Hip fractures are associated with loss of mobility and independence, reduced quality of life, and significant burden on the healthcare system [1]. Moreover, they are also linked to higher mortality rates compared to age-matched controls [2], with one-year mortality estimated as ranging from 15 to 36% [3]. Understanding the incidence of hip fractures and their subsequent complications is a vital first step in improving population health.

Numerous studies have reported seasonal variation in hip fracture incidence, with an increased prevalence in the winter [4–8]. This elevated risk during wintertime extends to countries with temperate and subtropical climates [9, 10], including Israel [11, 12]. Various explanations have been proposed for the increased risk of hip fracture in winter, such as adverse weather and outdoor conditions (e.g., slippery pavements), lower indoor and outdoor temperatures leading to hypothermia and impaired coordination, impaired vision due to winter darkness, and reduced Vitamin D levels. All these factors may contribute to an increased risk of falls, ultimately resulting in hip fracture [5–7].

However, contradictory findings exist. For instance, an Italian study spanning 4 years with a total of 8,978 cases reported no seasonal variation [13], while studies in Sweden [14] and Norway [15] were inconclusive regarding seasonal variation, possibly due to their reliance on data from individual years, making them susceptible to annual fluctuations. Another Norwegian study found seasonal variation only in fractures sustained outdoors [16]. Additionally, a recent Chinese study found that hip fractures were more likely to occur at higher temperatures than at lower temperatures, suggesting that older adults may prefer to stay indoors during extreme weather conditions, reducing the risk of falling [17]. Similarly, a Korean study noted an abrupt increase in the risk of hip fractures at temperatures exceeding 21 °C [18].

Several studies indicate that patient characteristics may influence the association between seasons and hip fracture incidence, though findings are contradictory. For instance, a Norwegian study by Solbakken et al. found the largest seasonal variations (difference between summer and winter) in men, in a younger age group (50–64), and in the healthiest group [7]. In contrast, Yee et al. demonstrated a higher incidence of hip fractures in winter in an older age group [8]. Moreover, a British study by Johnson et al. showed an association between lower temperatures and hip fractures in men but not in women [19]. Conversely, in a Chinese study, the associations between hip fractures and precipitation and higher temperatures were stronger in women than in men [17].

A few studies suggest that mortality following hip fracture may also vary by season, although the reasons for this are unclear. For example, a British study showed higher 30-day and one-year mortality for patients admitted in winter compared to those admitted in summer, though this was not adjusted for possible confounders and thus could not establish season per se as an independent risk factor for mortality [20]. Additionally, a recent British study observed higher 30-day mortality among patients presenting with a hip fracture in winter months, ranging from 6.7% in July to 8.7% (30% higher) in January [5]. Yee et al. found that hip fracture surgery performed in winter was associated with an increased risk of 30-day and 5-year mortality, even after adjusting for known risk factors affecting mortality [8].

Given these inconclusive findings, further investigation is warranted. Thus, the present study aims to explore seasonal variation in hip fracture incidence and mortality among older adults in Israel. This information may be instrumental in designing strategies to enhance population health.

## Materials and methods

### Study design

Retrospective study.

### Sample

This study included 400 elderly patients aged 65 and older, who were admitted with a hip fracture to a 495-bed hospital in northern-central Israel and underwent a hip fracture repair surgery during the years 2021–2022. Patients in this study received interdisciplinary treatment, involving coordinated care provided by multiple healthcare professionals from various disciplines, including orthopedic surgeons, geriatricians, nurses, physiotherapists, and social workers. This collaborative approach ensured comprehensive assessment, management, and rehabilitation tailored to individual patient needs. This study employed simple random sampling technique in selecting cases. Patients younger than 65 and patients with recurrent fractures were excluded.

### Data collection

The study was conducted after obtaining approval from the institutional Helsinki Committee. The data was collected retrospectively from the electronic medical records of hospitalized patients. The collected data included the following variables: gender, age, nationality, country of birth, place of residence, admission date, admission season, day of the week, Norton score, date of surgery, time of surgery, length of hospital stay, postoperative infection, type of infection, fall risk, readmission within 30 days, mortality, and date of death.

### Data analysis

Statistical analysis of the data was performed using SPSS software for Windows, version 27 (SPSS, Chicago, IL, USA). The seasons were classified as follows: Autumn - September, October, November; Winter - December, January, February; Spring - March, April, May; Summer - June, July, August. Chi-square tests (for categorical variables) and independent t-tests (for continuous variables) were used to identify risk factors for hip fracture. The chi-square test was used to investigate whether the season of admission is a risk factor for readmission and mortality. Kaplan-Meier survival curves were used to analyze survival probabilities among patients who underwent hip fracture repair in different seasons. Finally, Cox regression analysis was performed to identify predictors of mortality rate in the follow-up period (gender, age, nationality, and season of the year) among patients who underwent hip fracture repair. For all analyses, a significance level of *p* < 0.05 was considered statistically significant.

## Results

This study included 400 patients who had sustained a hip fracture in 2021 and 2022. This was a random sample from a total of 1,892 cases of hip fracture who were hospitalized and underwent a hip fracture repair surgery during these years. The sociodemographic characteristics of the sample are presented in Table 1. The mean age of the sample was 76.4 ± 10.8 years (range 65–107 years). The majority of the participants were female (75.5%). The most prevalent country of birth was Israel (54.3%), followed by “Other countries” (22%) and the former USSR (15.3%). The majority of the participants were Jewish (84.8%). Most participants were residing in urban areas (63.5%).

**Table 1:** Socio-demographic characteristics of the patients (*N* = 400)

Variable		M	SD	Range	%	*n*
Age		76.4	10.8	65–107		
Sex	Male				24.5%	98
	Female				75.5%	302
Country of Birth	Israel				54.3%	217
	Former USSR				%15.3	61
	Ethiopia				0.3%	1
	African countries				8.3%	33
	Other				22%	88
Ethnicity	Jewish				84.8%	339
	Arab				15.3%	61
Residence	City				63.5%	254
	Village				25%	100
	Kibbutz				5%	20
	Long-term care institution				6.5%	26
Month	January				8.5%	34
	February				7.2%	29
	March				13.3%	53
	April				9.5%	38
	May				8%	32
	June				7.2%	29
	July				7.5%	30
	August				7.2%	29
	September				10.5%	42
	October				4.5%	18
	November				8.0%	32
	December				8.5%	34
Season of the year	Autumn (September-November)				23%	92
	Winter (December-February)				24.2%	a
	Spring (March-May)				30.8%	123
	Summer (June-August)				22%	88
Day of the week	Sunday				9.8%	39
	Monday				17.3%	69
	Tuesday				16.5%	66
	Wednesday				15.3%	61
	Thursday				13.0%	52
	Friday				13.5%	54
	Saturday				14.8%	59
Norton score		12.6	2.2	7–19		
Risk of falls (Morse scale)		25.9	18.3	0–90		
Time from admission to surgery (in hours)		23.9	15.6	1–96		
Duration of hospital stay (in days)		7.0	6.2	2–84		
Infection following surgery	Yes				4.5%	18
Type of infection	COVID				3%	12
	ESBL				0.8%	3
	Scabies				0.5%	2
	VRE				0.3%	1
30-day readmission	Yes				11.3%	45
Died	Yes				20%	80
Life expectancy after surgery (in days) (deceased patients)		214.4	200.9	3-840		

The mean Norton score of the participants was 12.6 ± 2.2points (scores between 10 and 14 indicate a high risk for development of pressure ulcers). The mean fall risk score was 25.9 ± 18.3 (scores above 25 indicate a risk of falls).

Regarding the distribution of hip fracture cases throughout the months of the year, the highest number of cases was observed in March (13.3%) and the lowest in October (4.5%). When examining the distribution by season, there were more hip fracture cases in the spring (30.8%) than in other seasons (22-24.2%). Regarding the day of the week, a slight increase in hip fracture cases was observed at the beginning of the week (with 17.3% of cases occurring on Monday).

On average, participants underwent hip fracture repair surgery 24 ± 15.6 h after admission to the hospital (range 1–96 h). No significant difference was observed in preoperative time to surgery among different seasons (*p* > 0.05). The mean length of hospital stay was 7 ± 6.2days (range 1–96 days). Of all participants, 4.5% developed an infection during their hospital stay, with COVID being the most common type of infection. About 11% of participants were readmitted within 30 days. In total, 80 (20%) patients died, with a mean survival time of approximately 214 ± 200days (range 3-840 days).

In the present study, a correlation was found between sociodemographic characteristics and seasons of admission (Table 2). It was found that women were more likely than men to sustain a hip fracture in the spring (39.1% compared to 5.1%, respectively). On the other hand, men were more likely than women to experience hip fractures in the autumn (34.7% compared to 19.2%, respectively) and winter (32.7% compared to 21.5%, respectively). It is worth noting that among men, the lowest percentage of cases occurred in the spring (5.1%), while the percentage fluctuated around 30% in the other seasons. In contrast, among women, the highest percentage of cases occurred in the spring (39.1%), while the percentage fluctuated around 20% in the other seasons.

**Table 2:** Relationship between the socio-demographic characteristics and the seasons (*N* = 400)

Variable		Summer		Spring		Winter		Autumn		*P*-value
		%	*n*	%	*n*	%	*n*	%	*n*	
Gender	Male	27.6%	27	5.1%	5	32.7%	32	34.7%	34	0.01
	Female	20.2%	61	39.1%	118	21.5%	65	19.2%	58	
Country of Birth	Israel	12.4%	27	55.3%	120	14.7%	32	17.5%	38	0.01
	Former USSR	28.6%	20	17.1%	12	30.0%	21	25.7%	18	
	Africa	30.3%	10	3%	1	42.4%	14	24.2%	8	
	Other	34.1%	30	-	-	34.1%	30	31.8%	28	
Ethnicity	Jewish	21.5%	73	34.5%	117	23%	78	20.9%	71	0.01
	Arab	24.6%	15	9.8%	6	31.1%	19	34.4%	21	
**Variable**		**M**	**SD**	**M**	**SD**	**M**	**SD**	**M**	**SD**	**P-value**
Age		81.1	7.8	65.9	8.2	81.1	8.1	81.1	8.7	0.01
Duration of hospital stay (in days)		8.2	1.1	5.3	1.4	6.8	3.6	6.7	3.7	0.01

Furthermore, Israeli-born individuals were found to have a higher likelihood (compared to those born in other countries) of experiencing hip fractures in the spring (55.3% compared to 17.1% among former residents of the USSR). In contrast, Israeli-born individuals had a lower likelihood (compared to those born in other countries) of experiencing hip fractures in the winter (14.7% compared to 30.0% among former residents of the USSR). Additionally, in the summer and autumn, Israeli-born individuals had a lower likelihood (compared to those born in other countries) of experiencing hip fractures.

Moreover, Jews were found to have a higher likelihood than Arabs of experiencing hip fractures in the spring (34.5% compared to 9.8%, respectively). In contrast, Arabs had a higher likelihood than Jews of experiencing hip fractures in the autumn (34.4% compared to 20.9%, respectively).

Furthermore, differences in age and length of hospital stay were found among patients who experienced hip fractures in different seasons. Patients who experienced hip fractures in the spring were, on average, younger (M = 65.9 ± 8.2) than those who experienced hip fractures in other seasons (M = 81.1 ± 7.8–8.7). Additionally, the mean length of hospital stay for patients who experienced hip fractures in the spring was found to be the shortest (M = 5.3 ± 1.4), while the longest duration of hospital stay was observed in the summer (M = 8.2 ± 1.1).

**Fig. 1:**
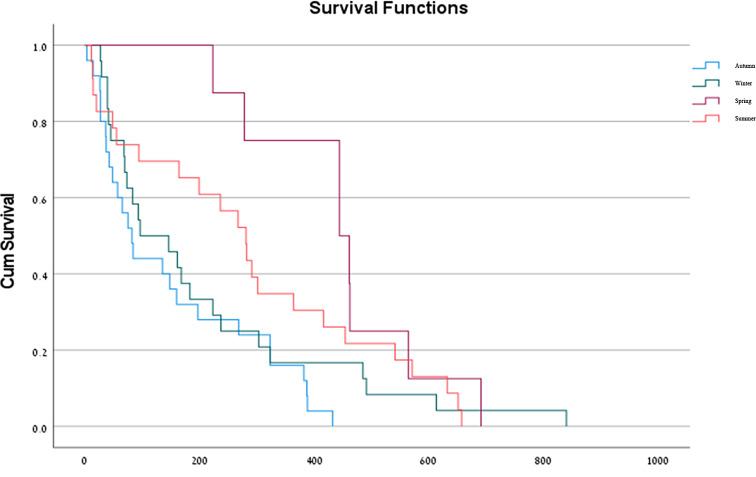
Kaplan-Meier curves: Seasonal variation in mortality following a hip fracture

A correlation between the seasons of the year and mortality rates was observed in the present study. Kaplan-Meier curves showed that patients who sustained a hip fracture in spring, had a higher probability of surviving one year after the hip fracture repair surgery (75%) in comparison with patients who sustained a hip fracture in the summer (30%), autumn (15%), and winter (15%) (Log rank *p* < 0.001) (Fig. 1). When entered into the multivariable Cox model, seasonality was found to be a significant independent predictor of mortality rate at the latest follow-up, after adjusting for gender, age, and nationality (HR = 12.09, 95% CI 94.95-241.04, *p* < 0.001). A regression analysis was conducted to investigate the association between patients’ age and mortality rates following hip fracture repair surgery. The analysis revealed a statistically significant relationship between patients’ age and mortality (χ^2^ = 367.203, *p* < 0.001). The Cox & Snell R Square and Nagelkerke R Square values indicated that age accounted for approximately 8.6–13.5% of the variance in mortality rates.

## Discussion

The aim of this study was to investigate seasonal variations in hip fracture incidence and mortality among older adults in Israel. Surprisingly, our findings indicate a higher incidence of hip fractures during the spring (30.8%) compared to other seasons, where the incidence was relatively consistent (22-24.2%). This unexpected pattern deviates from studies in various countries, including Israel [11, 12] which typically report increased hip fracture incidence during winter [4–8]. The inconsistency suggests a potential change in the activity levels of older adults in Israel, possibly influenced by the COVID-19 pandemic during 2021–2022. Older adults may have become more active following pandemic waves, consequently increasing their risk of falling and sustaining hip fractures. Notably, the higher incidence in spring, a comfortable season, does not rule out the role of weather conditions in hip fracture risk but does not strongly support it either.

In this study, individuals who fractured their hip in the spring were, on average, younger and had shorter hospital stays compared to those in other seasons. This implies that those experiencing hip fractures in spring were generally younger and in better health. These findings align with our hypothesis that the observed seasonal variation is linked to the activity levels of the elderly, with increased activity noted in the spring. These results resonate with a study by Solbakken et al., which found that the largest seasonal variations in hip fracture incidence occurred in the younger age group (50–64) and in the healthiest individuals, suggesting a correlation between physical activity and increased risk of falling [7].

Moreover, our study identified gender differences in hip fracture incidence, with a higher likelihood of hip fractures occurring in women during spring, and in men during autumn and winter. The lowest incidence among men was observed in spring (5.1%), while among women, there was a concentration of cases in spring (39.1%). These findings align with previous studies indicating gender-specific seasonal variations in hip fracture incidence [7, 17, 19]. Although the reasons for these gender differences remain unclear, our results suggest distinct patterns of activity between men and women across seasons, with women being more active in spring and men being less active during this season.

Furthermore, our study revealed significant variations in hip fracture incidence based on nationality and country of birth. Namely, Israelis had a higher likelihood of hip fractures in spring compared to non-Israelis (55.3% vs. 17.1%, respectively). Conversely, non-Israelis had a higher likelihood of hip fractures in winter compared to Israelis (30.0% vs. 14.7%, respectively). These disparities indicate a potential cultural influence on seasonal hip fracture patterns, possibly associated with various holidays observed in different communities.

Notably, no significant difference was observed in preoperative time to surgery among different seasons. As there is a mandated protocol in Israel to perform surgery within 24 h for patients with hip fractures, regardless of the season [20], this standardization likely accounts for the lack of variation in time to surgery observed across different seasons.

In terms of mortality, our study demonstrated a 20% mortality rate over the two-year follow-up period. While this proportion is lower than observed in a previous study in an Israeli cohort (30.0%) [21], it aligns with findings from an American cohort (23.5%) [22]. The differences may stem from methodological variations and distinct periods of data collection.

Moreover, our study revealed that patients who fractured their hip in autumn and winter had a lower life expectancy compared to those who sustained fractures in spring. This finding corroborates previous studies that reported higher mortality among patients presenting with hip fractures in winter months [5, 8, 23].

In our study, age accounted for approximately 8.6–13.5% of the variance in mortality rates, consistent with existing research showing that older age is associated with increased mortality following a hip fracture. This correlation underscores the physiological, social, and environmental factors contributing to higher mortality risk as individuals age. Physiological changes, prevalence of chronic illnesses, socioeconomic factors, and lifestyle choices all play significant roles in shaping mortality outcomes [24]. Importantly, seasonality emerged as an independent predictor of mortality after adjusting for gender, age, and nationality. This underscores the existence of seasonal variation in mortality following a hip fracture. The higher survival rate observed among patients who sustained a hip fracture in spring may be attributed to their overall better health status.

## Limitations

The study’s primary limitation lies in its single-institution focus, potentially limiting the generalizability of findings. Additionally, the reliance on data from only two consecutive years makes it susceptible to annual fluctuations. The retrospective nature of the chart review introduces the possibility of incomplete and inaccurate data, impacting the robustness of the results. Furthermore, the design of a retrospective study inherently precludes the establishment of causation.

## Conclusion

This study reveals a notable seasonal variation in hip fracture incidence among older adults in Israel, with a higher occurrence observed in the spring. This distinctive pattern may be attributed to potential shifts in the activity levels of older adults, possibly influenced by the global event of the COVID-19 pandemic. The findings highlight the substantial impact of broader societal factors on the incidence of hip fractures among older adults.

As individuals experiencing hip fractures in the spring appear to be in better health, our results underscores the importance of developing tailored preventive strategies specifically targeting this demographic, particularly through comprehensive patient education. This emphasis is particularly relevant for physically active older adults. In addition, our identification of seasonal variations in hip fracture incidence across demographic factors underscores the potential utility of the demographic factors in resource allocation and healthcare provider preparedness.

Our study supports the existence of seasonal variations in mortality following hip fractures, with the highest survival rates observed among patients sustaining fractures in the spring and the lowest in the autumn and winter. These patterns may be attributed to the better health status of patients who experience hip fractures in the spring. Consequently, healthcare organizations may have limited potential to alter this seasonal trend, emphasizing the importance of holistic patient care and addressing the broader societal influences on health outcomes.

While our study underscores notable seasonal fluctuations in hip fracture incidence and mortality rates among older adults in Israel, it is crucial to exercise caution when interpreting these findings regarding fracture prevention and post-fracture care. The increased incidence of hip fractures in spring, especially among younger patients with shorter hospital stays, highlights the necessity for deeper exploration into seasonal risk factors and tailored preventive strategies.

## Data Availability

No datasets were generated or analysed during the current study. The data that support the findings of this study are available from the corresponding author upon reasonable request.
